# Virome profiling of *Aedes albopictus* across urban ecosystems in Guangdong reveals sex-specific diversity

**DOI:** 10.1186/s13071-025-06872-2

**Published:** 2025-07-07

**Authors:** Qianlin Li, Jicheng Huang, Yanrong Zhou, Qin Wu, Jian Zhou, Fengxia He, Lirun He, Yongxia Shi, Cheng Guo, Jun Dai

**Affiliations:** 1 Health Inspection and Quarantine Laboratory, Guangzhou Customs Technology Center, Guangzhou, 510623 China; 2 School of Public Health, Sun Yat-Sen University, Guangzhou, 510080 China; 3 Guangdong International Travel Healthcare Center, Guangzhou, 510623 China; 4 Sichuan Center for Disease Control and Prevention, Chengdu, 610041 China

**Keywords:** *Aedes albopictus*, Mosquito, Next-generation sequencing, Guangdong, Diversity

## Abstract

**Background:**

*Aedes albopictus* mosquitoes are key vectors for arboviruses such as Dengue virus, Zika virus, and Chikungunya virus, posing significant global public health risks. Guangdong Province, a densely populated subtropical region in southern China, has experienced recurrent outbreaks of mosquito-borne diseases. However, sex- and geography-specific virome profiles of *Aedes albopictus* populations in this area remain uncharacterized, limiting the development of targeted surveillance strategies and precise risk assessment.

**Methods:**

We performed a metagenomic analysis of 1269 adult *Aedes albopictus* collected from five cities across Guangdong Province during autumn 2021. Mosquito pools underwent viral particle enrichment followed by DNA and RNA sequencing. Bioinformatic analyses were employed to characterize viral communities, evaluate alpha/beta diversity, and conduct phylogenetic reconstruction.

**Results:**

A comparative analysis of virome profiles in male and female *Aedes albopictus* across five regions of Guangdong Province (Chaozhou, Guangzhou, Shaoguan, Shenzhen, Zhanjiang) revealed significant viral distribution patterns influenced by both sex and geographic location. Female mosquitoes predominantly hosted vertebrate-associated arboviruses, including *Flavivirus*, consistent with their blood-feeding behavior. RNA virome composition showed significant sex-specific clustering (permutational multivariate analysis of variance, PERMANOVA, *P* = 0.008), with coastal cities (Shenzhen, Zhanjiang) being dominated by RNA viruses, whereas inland areas (Shaoguan) exhibited a predominance of DNA viruses.

DNA virome profiles displayed divergence between sexes but marked regional variation. Guangzhou emerged as an outlier, exhibiting exceptional bacteriophage diversity distinct from other regions. Phylogenetic analysis identified zoonotic pathogens with signatures of cross-species transmission and region-specific evolutionary adaptation. These findings highlight the interplay between mosquito ecology, geographic factors, and viral evolution in shaping virome diversity.

**Conclusions:**

This study presents the inaugural comparative analysis of DNA/RNA viromes in *Aedes albopictus* populations across Guangdong Province, revealing distinct sex-specific and geographic patterns in viral composition. The identification of vertebrate-associated viruses in female mosquitoes reinforces their epidemiological significance as arboviral vectors, while male-specific environmental viral signatures suggest potential pathways for ecological spillover. Coastal-inland and urban–rural disparities in viral communities emphasize the need for regionally tailored surveillance. These findings provide essential baseline virome data for forecasting emerging arboviral threats and informing strategies to mitigate zoonotic spillover in subtropical urban ecosystems.

**Graphical Abstract:**

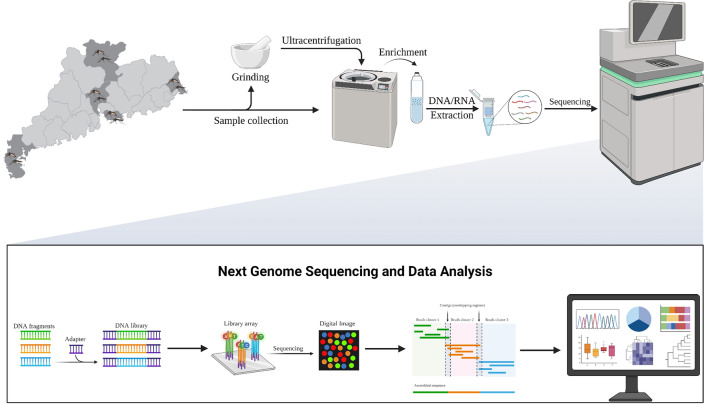

**Supplementary Information:**

The online version contains supplementary material available at 10.1186/s13071-025-06872-2.

## Background

Mosquitoes are primary vectors for several viral infections that remain a scourge of humanity. Members of the *Flaviviridae*, *Togaviridae*, and Bunyaviridae families, including several medically significant viruses, are predominantly transmitted by *Aedes* mosquitoes, which facilitate viral transmission between sylvatic cycles and mammalian hosts [1]. Current epidemiological data reveal the substantial burden of these pathogens: Japanese encephalitis virus (JEV) causes an estimated 50,000–175,000 human infections annually across Asia [2], while dengue virus (DENV) affects approximately 390 million people worldwide each year [3]. The global Zika virus (ZIKV) outbreak persisted through 2017 with active transmission reported in 64 countries and territories [4], and Chikungunya virus (CHIKV) reemerged to cause a 2019 epidemic along the China–Myanmar border [5]. Mosquito-borne viral diseases pose a major threat to human and animal health globally. Still, there are no available effective treatments against most of these viruses [6, 7]. Early and rapid detection of mosquito-borne diseases offers the potential to reduce the transmission risk.

*Aedes albopictus* (*Ae. albopictus*), originally from Southeast Asia, has emerged as a highly invasive mosquito species with global distribution [8]. In natural ecosystems, female *Ae. albopictus* mosquitoes serve as primary vectors for multiple arboviruses including DENV, ZIKV, CHIKV, and Yellow Fever virus (YFV), transmitting these pathogens through blood-feeding behavior [9]. Male adult *Ae. albopictus* mosquitoes feed primarily on plant-derived substances, which do not require blood meals for survival and reproduction, and it is unlikely to pick up pathogens back to hosts through bites [10]. However, human parechovirus (HpeV) genomic sequences were detected in the male mosquitoes [11]. Obtaining the HpeV from male *Ae. albopictus* might be by vertical transmission or in settings, such as contaminated aquatic environments [12]. Among these, the high possibility of environmental contact due to no evidence indicating that HPeV can be transmitted vertically. Hence, monitoring the virome of *Ae. albopictus* mosquitoes will reveal the status of circulating viruses in mammals and will be informative about both the abundance and the distribution of environmental viruses.

Guangdong Province, with its 126 million residents, represents China’s most densely populated region and a major economic hub. This subtropical coastal province’s unique combination of geographic proximity to Hong Kong and Macao, extensive trade networks, frequent air travel, and monsoon climate creates optimal conditions for arboviral transmission. *Ae. albopictus* dominates the mosquito population in Guangdong and has been implicated in recurrent arboviral outbreaks [13]. Surveillance data from 2005–2014 revealed that 94.3% of locally acquired DENV cases occurred in Guangzhou City [14], while Dongguan city reported China’s first indigenous CHIKV outbreak in 2010 [15]. Travel-associated Zika virus infections were subsequently documented in Guangzhou in 2016 [16]. These epidemiological patterns underscore the critical need for advanced viral surveillance systems in this high-risk region.

Next-generation sequencing (NGS) has revolutionized viral detection by enabling culture-independent analysis of viral communities [17, 18]. Viral metagenomics now facilitates comprehensive characterization of both RNA and DNA viruses across hosts, vectors, and environmental reservoirs, significantly expanding our understanding of viral diversity and distribution [19, 20]. Several novel viral sequences, including Cuacua virus, orbivirus, Castlerea virus, Viola virus, Zhejiang mosquito virus, and Wuhan mosquito virus, have been mapped in mosquitoes using this approach [21–24]. In addition, the most common mosquito-borne human viruses have also been detected, such as DENV, ZIKV, and West Nile virus (WNV) [25–27]. The diversity of mosquito-borne viruses likely contributes to the Global Virome Project. There is no previous report of viral composition and viral characteristics in *Ae. albopictus* female and male mosquitoes as assessed by NGS. Early detection of mosquito viral carriage not only dramatically expands the catalog of known viruses, but also is crucial for monitoring and controlling the emergence of arboviral disease.

Here, through integrated DNA and RNA viral metagenomics, we characterized virome profiles in *Ae. albopictus* populations across ecologically distinct urban areas of Guangdong Province, China—a critical subtropical region for arboviral transmission. Analyzing mosquitoes from five cities revealed fundamental sex- and geography-dependent viral partitioning: females exhibited greater carriage of vertebrate-associated arboviruses correlated with hematophagic behavior, contrasting with male-enriched plant/environment-associated viruses. While DNA viromes demonstrated predominant geographic differentiation with exceptional diversity observed in Guangzhou, RNA viromes showed pronounced sex-specific clustering and coastal-inland divergence. Our analysis identified 17 viral families including emerging zoonotic pathogens and novel unclassified viruses, with phylogenetic evidence suggesting localized viral evolution and cross-species transmission potential. Notably, coastal populations displayed RNA-dominated viral communities versus DNA-dominated profiles inland. These findings establish essential baseline data on mosquito viromes in a strategic border region, highlighting surveillance priorities for arboviral spillover risks in urban ecosystems.

## Methods

### Mosquito sampling and pooling information

**Fig. 1:**
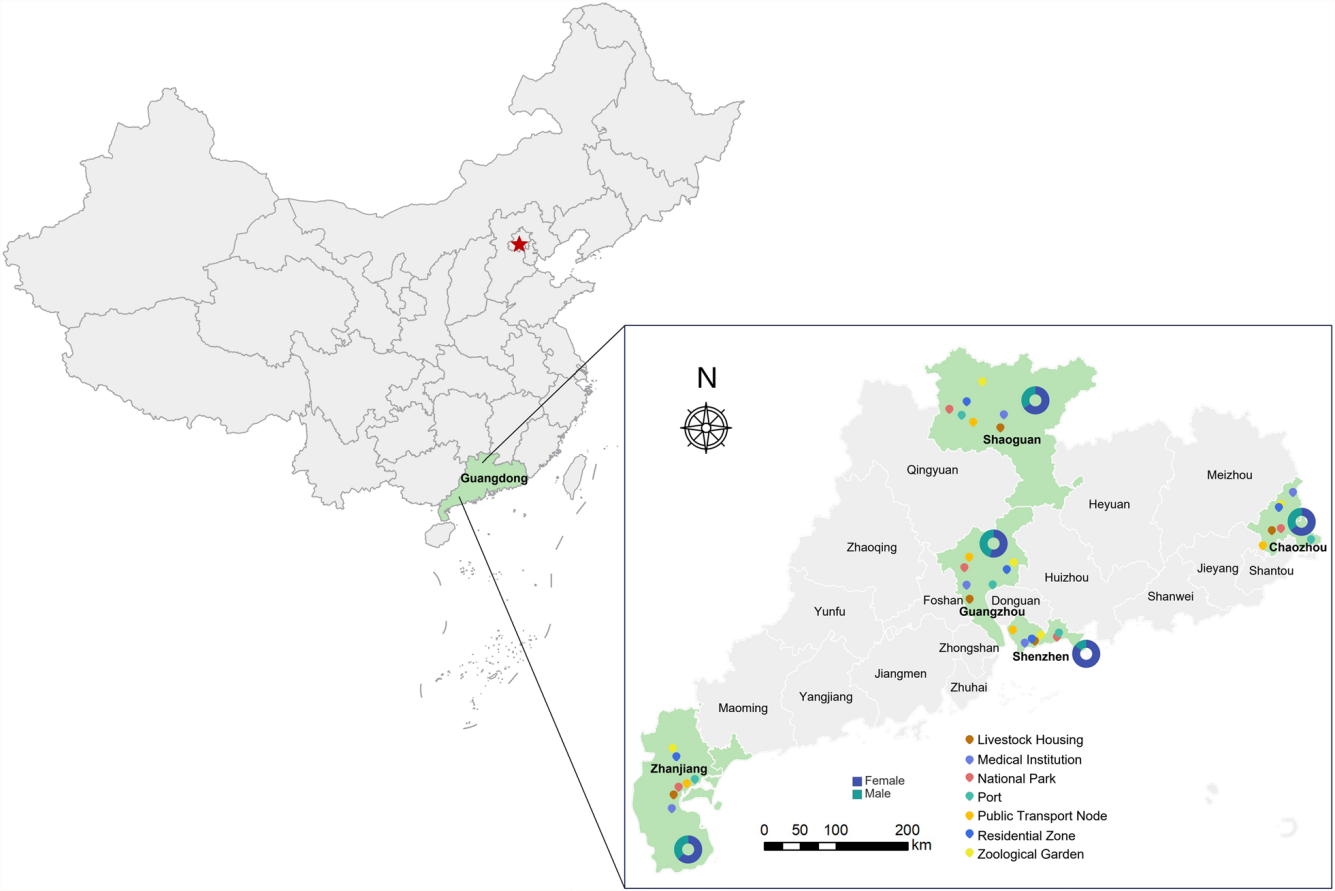
Geospatial mapping of sex-specific collections of *Aedes albopictus* mosquitoes in major cities of Guangdong Province, China

Adult *Ae. albopictus* mosquitoes were systematically collected during autumn 2021 (September–November) across 35 sampling sites in Guangdong Province, China as illustrated in Fig. 1 (geographical distribution map), encompassing seven distinct habitat types: livestock housing, medical institutions, national parks, ports, public transport nodes, residential zones, and zoological gardens. Field collections were conducted in five representative cities (Chaozhou, CZ; Guangzhou, GZ; Shaoguan, SG; Shenzhen, SZ; Zhanjiang, ZJ) using standardized hand-held aspirators.

Captured specimens underwent rigorous morphological identification through stereomicroscopic examination of taxonomically diagnostic characteristics, including cephalic structures (antennal morphology and maxillary palp features), thoracic scutal patterns, abdominal markings, wing venation architecture, and leg banding coloration, following established entomological keys. Males exhibited characteristically elongated, plumose palps contrasting with the shorter, proboscis-length palps in females. Post-collection processing involved immediate euthanasia via controlled freezing (−20 °C) in portable refrigeration units to maintain biomolecular integrity. Specimens were subsequently cryopreserved in dry ice-cooled insulated containers during transportation to ensure optimal condition preservation for virological analyses.

The collected mosquitoes were systematically pooled according to sex and geographic origin, with female cohorts (CZF, GZF, SGF, SZF, ZJF) containing 100~200 individuals per pool, and male groups (CZM, GZM, SGM, SZM, ZJM) comprising 20~200 specimens per pool. All procedures involving potentially infectious materials were conducted in compliance with biosafety protocols within certified Biosafety Level 2 containment facilities.

### Sample preparation and enrichment

For sample preparation, a pivotal step is the enrichment of viral particles (VLPs) from samples containing vast microbial constituencies. The VLPs of mosquito pools were prepared as follows. Briefly, mosquito tissues were harvested and ground in liquid nitrogen. Virus-containing supernatants were centrifugation at 12,000 × g for 5 min at 4 °C and then passed through 0.45 µm and 0.22 µm filters. The supernatants were gently layered on top of the 28% sucrose solution and centrifuged at 160,000 × g for 2 h at 4 °C. SM buffer (100 mM NaCl, 8 mM MgSO_4_, 50 mM Tris, pH 7.5) containing the pellet viral particles was collected and 10 × DNaseI was added. After incubation at 37 °C for 60 min, EDTA was added to a concentration of 5 mM to stop the reaction by incubating the reaction at 65 °C for 10 min. Conditioned supernatant was finally centrifuged at 2000 rpm for 5 min at 4 °C to get VLPs. 

### DNA and RNA extraction

The total viral nucleic acid in the obtained VLPs was isolated using MiniBEST Viral RNA/DNA Extraction Kit Ver.5.0 (Takara, Japan) according to the manufacturer’s instructions. Extracted DNA and RNA were subjected to whole genome amplification (Ready-To-Go GenomiPhi V3 DNA Amplification Kit, GE Healthcare, USA) and whole transcriptome amplification (REPLI-g Kit, Qiagen, Germany) to increase template concentration, respectively. DNA fragment purity and concentration were determined using a Nanodrop spectrophotometer (Thermo Fisher Scientific, USA) for initial assessment and the Qubit High Sensitivity DNA assay (Life Technologies, USA) for accurate quantification, with subsequent fragment size verification performed via 1% agarose gel electrophoresis.

### Library construction and high-throughput sequencing

Sequencing libraries were constructed separately for DNA and RNA viruses using the NEBNext Ultra II kit. Qualified DNA or cDNA library was constructed with the Ultra™ II DNA Library Prep Kit for Illumina (E7645S, NEB) according to the manual. Paired-end sequencing was used in the Illumina HiSeq2500 platform with a 150 bp read length at MEGIGENE Biological Company in Guangdong, China.

### Bioinformatic and statistical analysis

Raw FASTQ sequencing data were analyzed through CZID, a web-hosted, open-source bioinformatics system designed for microbial community characterization using metagenomic sequencing information. This computational platform is publicly accessible at http://czid.org/ [28]. Briefly, the processing pipeline was as follows: (1) raw data quality control: sequencing adapters, short reads (< 35 bp), low-quality bases (Phred score < 17), reads with > 15 undetermined bases (Ns), and low-complexity regions (> 40% repeats) were filtered using fastp, generating QC-filtered reads; (2) host DNA removal: QC-filtered reads were aligned to the *Ae. albopictus* reference genome using STAR and Bowtie2 [29] to exclude host-derived sequences, yielding clean reads for virome analysis; (3) human read removal: removal of human sequences identified through Bowtie2 followed by HISAT2 alignments against reference human genomes; and (4) viral assembly: genomic sequences were reconstructed through de novo assembly of processed reads using SPAdes; assembly quality metrics including contig integrity and sequencing depth were verified through Bowtie2-based read alignment. Viral-associated contigs were subsequently identified through comprehensive BLASTN (nt database) and BLASTX (nr database) analyses to confirm viral origin and validate the consensus genomes.

Mosquito-associated viral sequences were identified by applying the following threshold filters: *Z*-score > 1, Nt rpM ≥ 10, and Nt contigs ≥ 1 [30, 31]. Data were normalized to unique Nt rpM input reads for each virome at both species and genus levels. To assess alpha-diversity of the viral communities, the Shannon index was used, and beta-diversity analysis was conducted using Bray–Curtis distance and PERMANOVA for significance testing. Species that showed significant differences between female and male groups were identified using the LDA Effect Size (LEfSe) method [32]. Comparisons between two groups were made using the nonparametric Mann–Whitney *U* test. For all analyses, significant findings were defined as *P* ≤ 0.05. The evolutionary tree of viral sequences was constructed using MEGA with the neighbor-joining. Visualization was achieved using the ggplot2 R package.

## Results

### Comparative metagenomic analysis of DNA and RNA viruses in *Ae. albopictus*

The diverse urban and periurban ecosystems of Guangdong Province, characterized by densely populated residential areas, transportation hubs (e.g., airports and ports), and heterogeneous environments (parks, hospitals, zoos), provide favorable habitats for *Aedes albopictus*, a major vector for arboviruses such as dengue, Zika, and Chikungunya. To investigate the composition and diversity of DNA and RNA viruses harbored by this invasive mosquito species across ecologically distinct settings, we conducted a large-scale collection of *Ae. albopictus* in September 2021 from five cities in Guangdong (Guangzhou, Shenzhen, Shaoguan, Zhanjiang, and Chaozhou), a region bordering greater bay area with frequent international travel and trade (Fig. 1). Adult mosquitoes collected from multiple cities were pooled into 20 sex-specific groups (10 female and 10 male), with each pool containing 20~200 individuals homogenized across collection sites.

**Fig. 2:**
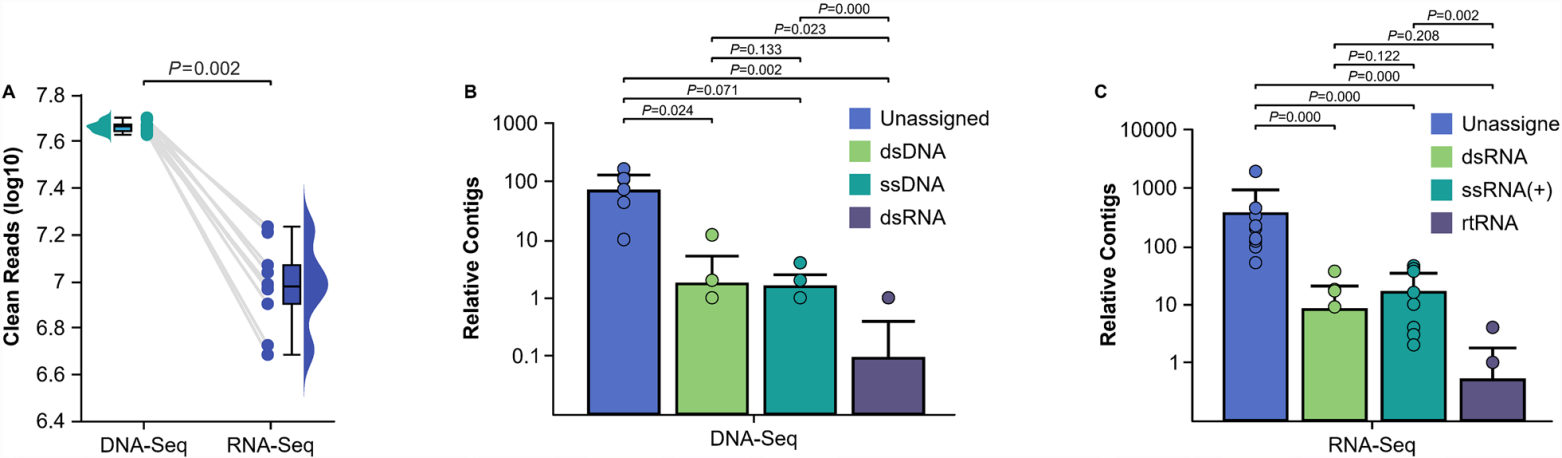
Comparative analysis of clean read distributions and viral contig abundance in DNA-Seq versus RNA-Seq. **A** Significant differences in clean reads between DNA-Seq and RNA-Seq. **B** Profiling the compositional types of viral DNA. **C** Profiling the compositional types of viral RNA

We generated approximately 1.23 billion paired-end reads (150 bp) from the 20 pools, including 661 million reads for DNA viromes and 564 million reads for RNA viromes (Supplementary Tables 1 and 2). After stringent filtering of host-derived sequences, 892 million clean reads (DNA: 460 million; RNA: 102 million) were retained for viral metagenomic analysis (Supplementary Tables 1 and 2). Comparative analysis of DNA-Seq and RNA-Seq data revealed a statistically significant difference (*P* = 0.002) in the total clean reads between the two groups (Fig. 2A). Both DNA-Seq (Fig. 2B) and RNA-Seq (Fig. 2C) analyses revealed a predominant proportion of unclassified sequences, suggesting the existence of novel viral species or potential interference from host-derived symbiotic microorganisms. We identified 17 viral families, including both arthropod-specific viruses (e.g., Alphatetraviridae, Mesoniviridae, Permutotetraviridae, and Phasmaviridae) and vertebrate-infecting arboviruses (e.g., Anelloviridae, Flaviviridae, Parvoviridae, and Retroviridae) (Supplementary Figs. 1 and 2).

### Sex- and geography-specific virome profiles of *Aedes albopictus*

**Fig. 3:**
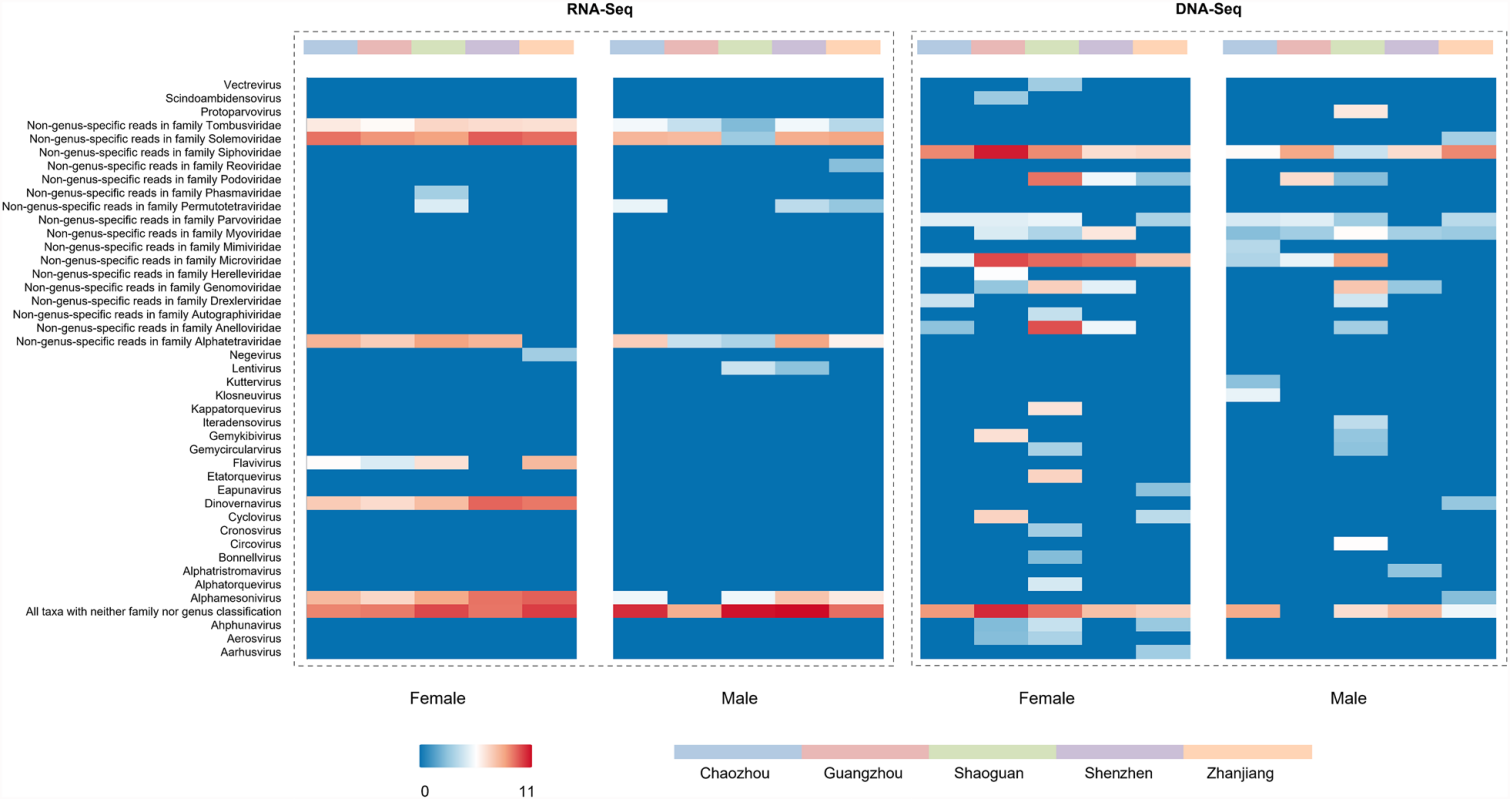
Sex- and geography -resolved virome profiles of *Aedes albopictus* in Guangdong, China. Bars represent viral read abundance from DNA-Seq and RNA-Seq, categorized by genus or nonspecific classifications

The virome analysis of *Ae. albopictus* populations across five regions in Guangdong Province demonstrated distinct sex- and region-dependent variations in viral composition (Fig. 3). DNA-Seq and RNA-Seq uncovered 43 unique viral genera, including numerous non-genus-specific viral reads (Supplementary Figs. 3, 4). RNA-Seq analysis revealed Flavivirus and Dinovernavirus were commonly detected in female mosquito samples across multiple locations, suggesting that the *Ae. albopictus* populations in these areas may harbor high viral loads. Male mosquitoes exhibited restricted RNA viral activity, with Lentivirus showing unique enrichment specifically in populations from Shaoguan and Shenzhen. Non-genus-specific RNA virus families, such as Solemoviridae and Tombusviridae, were broadly distributed across female and male samples, with Shenzhen females showing the highest unclassified Solemoviridae reads. DNA-Seq analyses revealed pronounced disparities in virome composition between sexes and geographic regions. Female and male mosquitoes exhibited marked enrichment of bacteriophage-associated taxa, including Microviridae, Siphoviridae, Myoviridae, and Podoviridae.

DNA-Seq and RNA-Seq analysis revealed distinct geographical variations in viral genus distribution across the surveyed regions. All taxa with neither family nor genus classification demonstrated predominant prevalence in all areas, with Guangzhou exhibiting exceptionally high viral loads, surpassing other regions by 2–3 orders of magnitude. Shenzhen demonstrated the highest relative abundance of non-genus-specific Solemoviridae and Dinovernavirus in RNA-Seq analyses, whereas Guangzhou showed predominance in DNA-Seq-specific non-genus-specific Siphoviridae and Microviridae reads. Shaoguan exhibited the broadest spectrum of DNA-Seq-detected viral types, forming a striking contrast with Shenzhen’s consistently lowest detection rates across most taxa. Non-genus-specific read distribution followed a clear geographical divergence, with coastal cities such as Shenzhen and Zhanjiang showing RNA-Seq predominance and inland regions such as Shaoguan displaying DNA-Seq dominance.

### Sex-specific alpha-and beta-diversity patterns in DNA-Seq and RNA-Seq

**Fig. 4:**
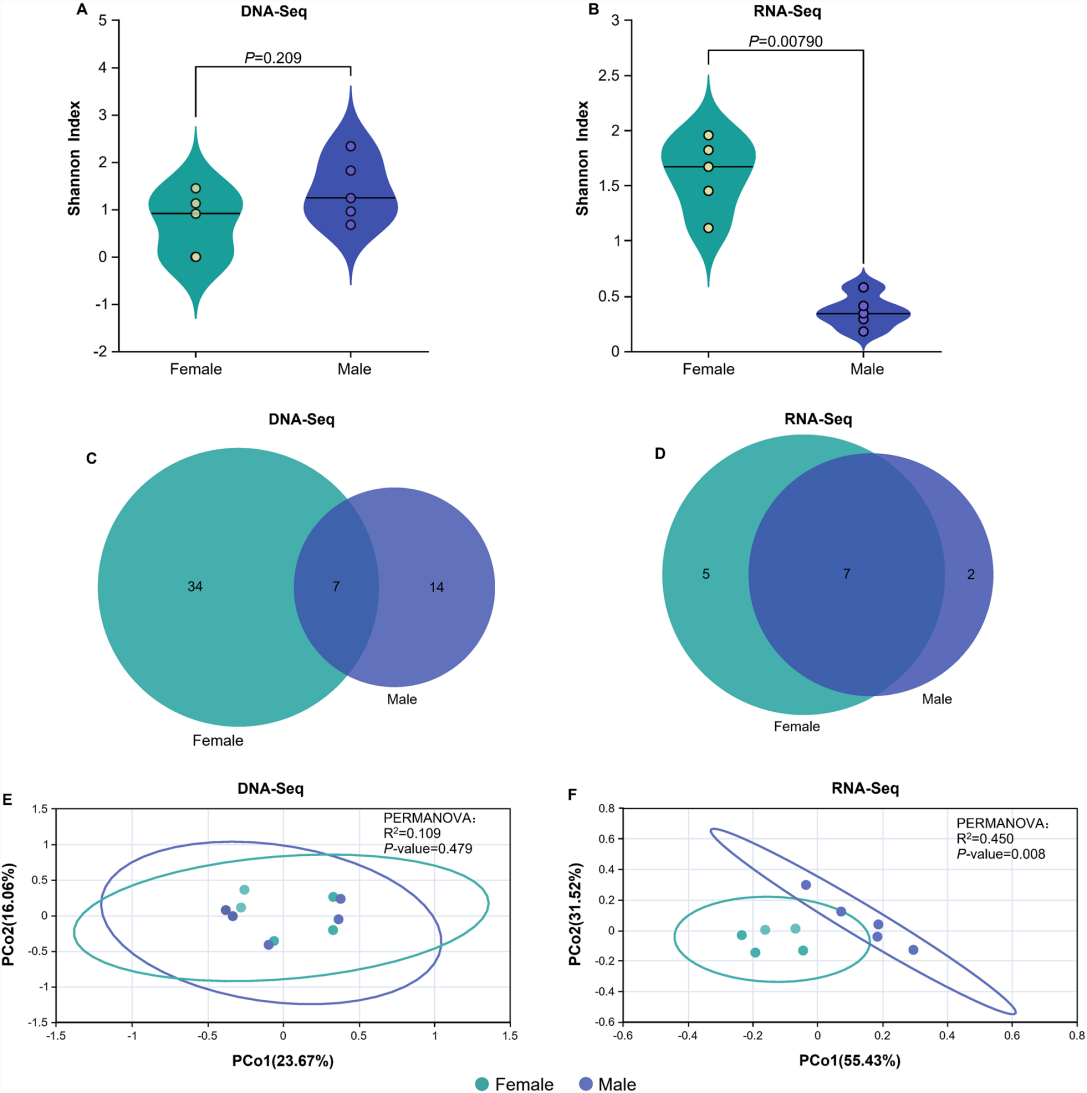
Sex-specific alpha- and beta-diversity patterns in viral community profiling. **A** Sex-specific alpha diversity variation in DNA-Seq community. **B** Sex-specific alpha diversity variation in RNA-Seq community. **C** Venn diagram of male and female mosquito virus species in DNA-Seq. **D** Venn diagram of male and female mosquito virus species in RNA-Seq. **E** Principal coordinate analysis (PCoA) plot of the beta-diversity in DNA-Seq. **F** PCoA plot of the beta-diversity in RNA-Seq

DNA-Seq analysis demonstrated no statistically significant alteration in microbial α-diversity between sexes (Fig. 4A), as evidenced by the extensive overlap in Shannon index distributions (*P* = 0.209). This indicates that host sex minimally influences taxonomic composition diversity at the genomic DNA level. But RNA-seq data (Fig. 4B) revealed pronounced sex-specific divergence (*P* = 0.0079), with males exhibiting broader dispersion in Shannon index variability compared with the tightly clustered distribution pattern observed in females. In addition, Chaozhou and Shenzhen exhibit near-equilibrium contributions, while Shaoguan shows pronounced female dominance. This suggests that sex-specific disparities are environment-dependent and influenced by sampling location (Supplementary Table 3).

A notable sex-based disparity exists between male and female mosquito virus groups concerning DNA viruses: females harbor 34 unique viruses, while males possess 14 unique viruses, with only 7 viruses being common to both sexes (Fig. 4C). Conversely, the findings related to RNA viruses revealed an inverse pattern: the number of shared viruses between males and females remained consistent at seven, yet the number of sex-specific viruses exhibited a marked decline, with five unique to females and two unique to males (Fig. 4D).

The DNA-specific beta-diversity divergence revealed minimal separation between female and male microbial communities, with PCo1 and PCo2 explaining only 23.67% and 16.06% of total variance, respectively. The overlapping data points and nonsignificant permutational multivariate analysis of variance (PERMANOVA) result (*R*^2^ = 0.109, *P* = 0.479) confirm that host sex does not significantly influence taxonomic structure at the genomic DNA level (Fig. 4E). Conversely, RNA-Seq data (Fig. 4F) exhibited pronounced sex-specific clustering, with PCo1 and PCo2 accounting for 55.43% and 31.52% of variance, respectively. The clear separation between sexes in RNA-Seq, supported by strong PERMANOVA significance (*R*^2^ = 0.450, *P* = 0.008), highlights sex-dependent functional divergence in transcriptionally active microbial communities.

### Sex-driven distribution of arboviral viruses in *Aedes albopictus*

**Fig. 5:**
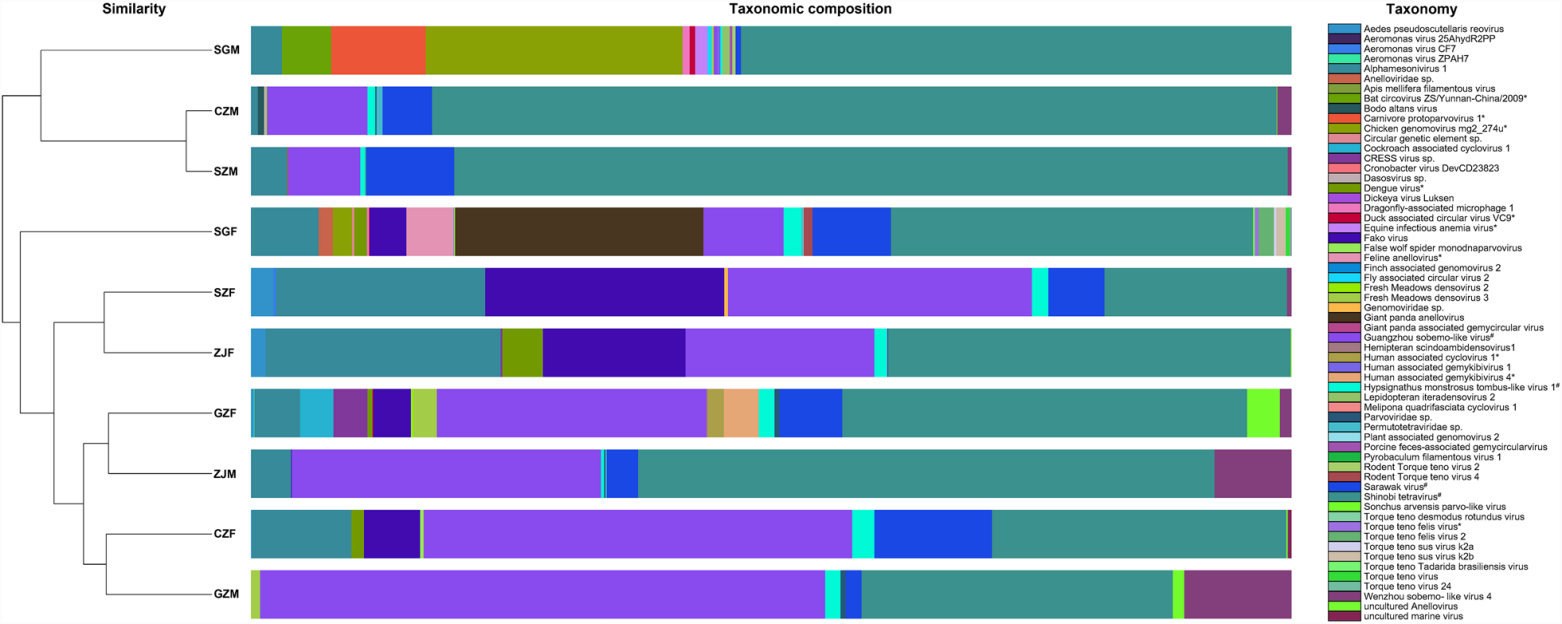
Taxonomic profiling and hierarchical clustering of viral communities across diverse sample types. Abbreviations: Chaozhou, CZF/CZM; Guangzhou, GZF/GZM; Shaoguan, SGF/SGM; Shenzhen, SZF/SZM; Zhanjiang, ZJF/ZJM. * and ^#^ symbols represent vertebrate-associated viruses and represent non-sex-specific viruses, respectively

Samples exhibited strong regional clustering (Fig. 5), with mosquitoes from SGF and SGM harboring a high proportion of chicken genome virus mg_2274u, suggesting a close association with local livestock or poultry farming activities. Furthermore, ZFN specimens clustered closely due to the dominance of Torque teno virus variants, which are widespread in mammals, indicating cross-species transmission risks via mosquito-livestock interactions. Female mosquitoes from GZF carried higher abundances of zoonotic pathogens, including human-associated cyclovirus 1 and human-associated gemykibivirus 4, consistent with their hematophagous behavior and frequent mammalian host contact. Male mosquitoes from CZM and GZM predominantly hosted plant/environment-associated viruses (Sonchusarvensis parvo-like virus, Cressdnaviricota sp), reflecting their reliance on plant sap feeding. The widespread presence of Shinobi tetravirus, Sarawak virus, Hypsignathus monstrosus tombus-like virus 1, and Guangzhou sobemo-like virus in both sexes indicates non-sex-specific infections, likely due to common ecological or physiological characteristics.

**Fig. 6:**
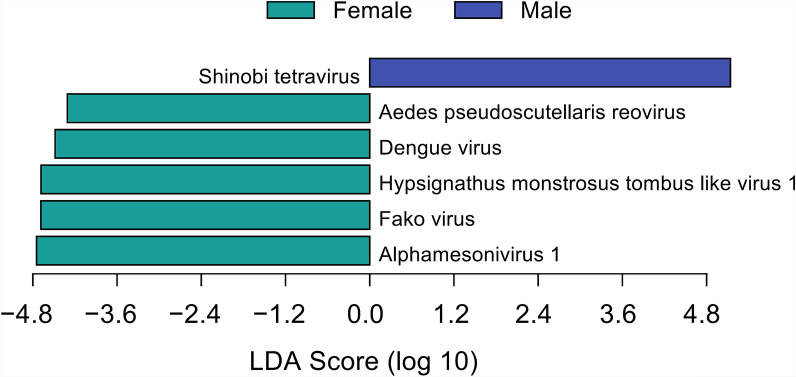
Sex-based differential abundance of mosquito-borne viruses identified via LEfSe analysis

The LDA of sex-specific viral loads in mosquitoes revealed distinct sex-based virome profiles (Fig. 6). Shinobi tetravirus demonstrated the highest degree of male tropism, with a LDA score of 5.14, followed by Alphamesonivirus 1 with an LDA score of 4.75. In addition, Fako virus (LDA = 4.69) and Hypsignathus monstrosus tombus-like virus 1 (LDA = 4.68) showed moderate specificity toward females. DENV, which is predominantly transmitted by blood-feeding female vectors, had an LDA score of 4.48.

### Phylogenetic analysis of vertebrate-associated viruses

**Fig. 7:**
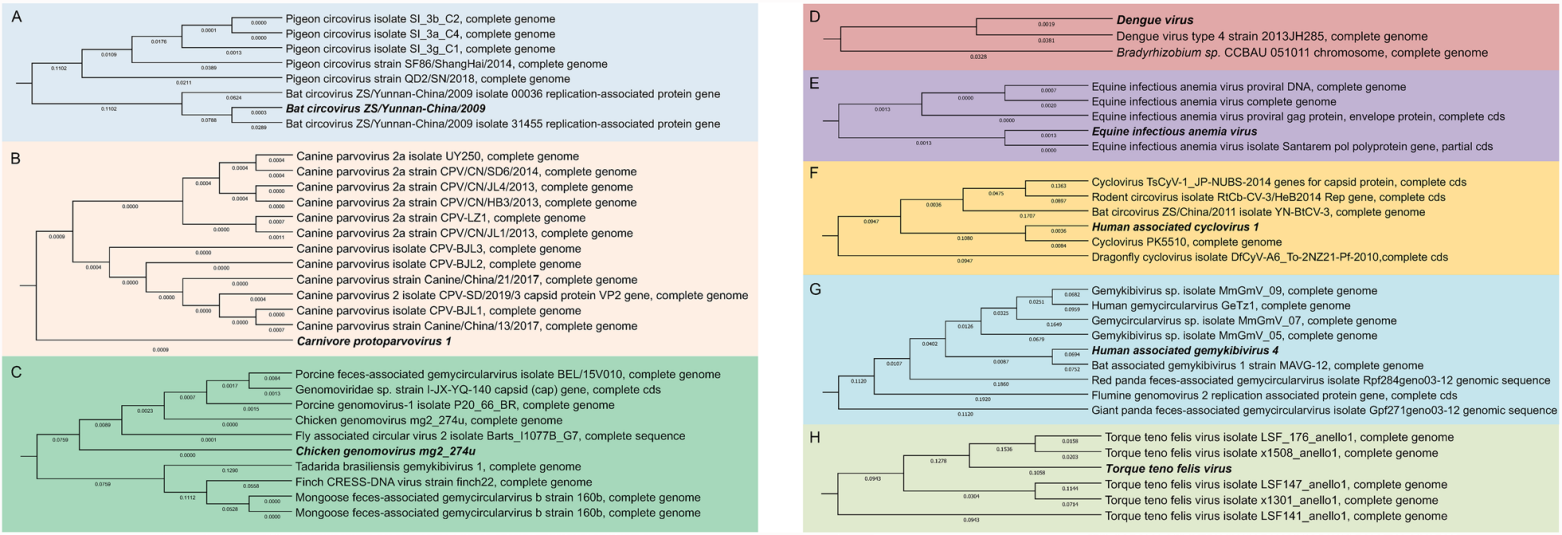
Phylogenetic classification of viral genomes. (A–H) Phylogenetic trees of vertebrate-associated viruses: *Bat circovirus* (**A**), *Carnivore protoparvovirus* 1 (**B**), *Chicken genomovirus mg2_274u* (**C**), *dengue virus* (**D**), *Equine infectious anemia virus* (**E**), *Human associated cyclovirus 1* (**F**), *Human associated gemykibivirus 4* (**G**), and *Torque teno felis virus* (**H**)

We identified the sequences of ten viruses associated with vertebrates (Supplementary Table 4 and Fig. 7A–H). Circoviruses exhibit distinct evolutionary clusters in birds and mammals, as revealed by phylogenetic analysis (Fig. 7A). The virus shares a primary clade with Bat circovirus ZS/Yunnan-China/2009, exhibiting a remarkably short genetic distance (branch length) of 0.0788, which is significantly smaller than that observed with Pigeon circovirus strain. These findings suggest that Bat circovirus isolate may represent the common ancestor of this virus, with potential transmission occurring through host-jumping mechanisms.

As shown in Fig. 7B, the phylogenetic analysis of canine parvovirus (CPV) strains reveals different evolutionary clustering patterns among regional isolates. The tree uses the carnivorous prototype parvovirus 1 as an outgroup, indicating that CPV 2a strains (such as UY250, CPV/CN/SD6/2014, CPV/CN/JL4/2013) form a monophyletic branch with high node support, suggesting that they have a conserved genome structure and a common evolutionary trajectory. It is worth noting that Chinese isolates from 2013 to 2019 clustered into the smallest subclass of branch length (≤ 0.0009 substitutions/sites), indicating local viral diversity and sustained transmission in the region.

The comparative genomic analysis presented in Fig. 7D corroborates the presence of DENV-4 2013JH285 in mosquito samples; however, the exceedingly low genomic coverage limits the ability to draw definitive evolutionary or functional inferences. Moreover, the minimal similarity to *Bradyrhizobium* sp. highlights the specificity of the viral signal. The phylogenetic tree of Equine infectious anemia virus displayed minimal genetic variation (branch length: 0.0000–0.0020), suggesting a monophyletic origin with little subtype diversity (Fig. 7E).

Figure 7F revealed that bat circovirus YN-BtCV-3 displayed closer evolutionary affinity to human-associated cyclovirus 1, suggesting potential cross-species transmission or conserved genomic features between bat and human-associated lineages. MmGmV_07 and MmGmV_05 showed close affinity to human-associated gemykibivirus 4, implying potential host-switching events between mammalian hosts (Fig. 7G).

Phylogenetic analysis of Torque teno felis virus isolates (LSF_176_anello1, x1508_anello1, and LSF141_anello1) showed varied evolutionary patterns and genetic diversity (Fig. 7H). The tree structure revealed significant branch length changes, with x1508_anello1 having the longest branch (0.1536), suggesting geographic isolation or host adaptation may speed up differentiation. Closely related isolates such as LSF141_anello1 and LSF147-anello1 (branch length 0.0304) formed clusters with high node support (0.0943–0.1144), indicating conserved genomic evolution in certain lineages. 

## Discussion

Guangdong Province’s tropical climate fosters optimal conditions for mosquito vector proliferation and arboviral transmission, with *Ae. albopictus* serving as a key bridge vector for multiple pathogens. Notably, *Ae. albopictus* mosquitoes transmit multiple pathogenic arboviruses including DENV, ZIKV, and JEV [33]. Furthermore, studies have identified variations in the genetic diversity and population structure of *Ae. albopictus* across various regions in China. This genetic diversity is potentially linked to the mosquitoes’ resistance to insecticides, particularly in the southern regions where *Ae. albopictus* exhibits greater genetic diversity. Such diversity may influence the species’ capacity to transmit viruses [34, 35]. This study investigates sex- and geography-related viral diversity through comprehensive virome characterization of *Ae. albopictus* populations across Guangdong’s subtropical urban ecosystems, providing a scientific foundation for the monitoring and prevention of mosquito-borne viral diseases within subtropical urban ecosystems.

Sex-specific virome profiles observed in *Ae. albopictus* populations from Guangdong Province demonstrate distinct patterns correlating with biological and ecological dimorphism between sexes. Female mosquitoes exhibited higher RNA viral activity, particularly with Flavivirus and Dinovernavirus, which may reflect their hematophagous behavior and role as primary vectors for arboviral transmission [36]. These findings are consistent with studies highlighting female-specific blood-feeding requirements for oogenesis, which increase exposure to vertebrate-associated viruses [37]. Males exhibited comparatively reduced RNA viral diversity, potentially associated with their phytophagous feeding behavior and environmental microbial interactions [38]. Non-host-specific RNA viral families including Solemoviridae and Tombusviridae, showed ubiquitous distribution across both sexes, suggesting possible environmental acquisition through shared horizontal transmission mechanisms. DNA virome analysis revealed bacteriophage predominance, with Microviridae and Siphoviridae constituting major components. This phage prevalence highlights complex host-microbiota interactions influencing mosquito physiology, potentially modulated by sex-dependent immune regulation or microbiome compositional variations [36, 39].

Geographical differences in viral composition may reflect both ecological and methodological influences. Coastal cities such as Shenzhen and Zhanjiang showed a dominance of RNA viruses, which could be partially attributed to subtropical climates favoring their persistence in aquatic or human-altered environments [40]. However, methodological factors, such as potential RNA degradation during sample transport from humid coastal areas or region-specific sequencing focus, cannot be ruled out. Conversely, inland areas such as Shaoguan had more DNA viruses, potentially indicating stable bacteriophage communities in less urbanized regions with varied larval habitats [41], though the observed DNA-Seq dominance might also relate to standardized protocols for DNA virus preservation. Further studies controlling for technical variables are needed to disentangle ecological and methodological contributions. RNA-Seq analysis (PCo1 + PCo2) explained 86.95% of the variance, significantly more than DNA-Seq’s 39.73%, showing RNA data better capture biological differences. This is supported by RNA-Seq’s 4.1-fold higher *R*^2^ value (0.450 versus 0.109), highlighting that sex-related microbial differences are mainly seen in transcriptional activity, not taxonomy.

Our findings have demonstrated that the regional clustering patterns of the mosquito virome are strongly correlated with local agricultural practices. The pronounced prevalence of chicken-associated viruses in SGF and SGM samples highlights their close connection between the virome and livestock farming activities, consistent with the hypothesis that mosquito host phylogenetics and environmental factors (such as farm proximity) drive virus diversity [42, 43]. In addition, the high proportion of circovirus variants in ZFN samples reveals the risk of cross species virus transmission in areas with high human animal contact, especially in intensive farming areas where comprehensive monitoring needs to be strengthened.

The study also revealed the ecological mechanism of sex-specific viral differences in mosquitoes. Female mosquitoes carry more zoonotic pathogens (such as human-associated cyclovirus 1 and human-associated gemykibivirus 4) due to their blood sucking behavior, which is consistent with their frequent contact with mammalian hosts; male mosquitoes feed on plant sap, and their viral repertoire is mainly composed of plant/environment related viruses (such as Sonchusarvensis parvo-like virus). The moderate specificity of vector specific pathogens such as DENV in female mosquitoes (LDA = 4.48) confirms the classic theory that female mosquitoes are the main vector of transmission. However, the presence of non-sex-specific viruses such as Sarawak virus suggests that symbiotic environments or immune tolerance may promote transgender transmission of the virus [44].

Previous viromics studies on *Ae. albopictus* in Guangzhou have identified several known viruses, including Wenzhou sobemo-like virus 4, mosquito nodular virus, Aedes yellow fever virus, Hubei chryso-like virus 1, and tobacco rattlesnake virus RNA1. Additionally, 21 novel viruses previously unreported were discovered, whose diversity and genomic characteristics offer valuable new insights [45]. DENV has been found in several regions, posing a potential risk of dengue fever transmission. Confirmation alongside mosquito species identification is required. Notably, the presence of TTFV in female mosquitoes indicates a possible expansion of host range or mechanical transmission, though further validation of virus replication in mosquitoes is needed. The genetic distance (0.0158–0.1536) and varied branch support (e.g., 0.1278 for x1508anello1) suggest recombination or adaptive mutations in key genomic areas such as ORF1. These findings reveal notable differences in the viral spectrum of *Ae. albopictus* across regions and genders, potentially impacting virus transmission and control strategies [46, 47].

## Conclusions

This study highlights the dual utility of sex-specific and geography-stratified virome analysis in *Aedes albopictus*, a species of growing public health concern due to its expanding global range. The findings provide critical baseline data on understudied DNA/RNA viral communities in a key border region, offering insights into potential spillover risks and informing targeted surveillance strategies for emerging arboviral threats in subtropical urban ecosystems.

## Supplementary Information

Supplementary material 1.

Supplementary material 2.

## Data Availability

No datasets were generated or analyzed during the current study.
