# Supplementary material for: Virome profiling of Aedes albopictus across urban ecosystems in Guangdong reveals sex-specific diversity
Source: Parasit Vectors. 2025 Jul 7;18:264. doi: 10.1186/s13071-025-06872-2 (PMC12236024; doi:10.1186/s13071-025-06872-2)
Supplement: Supplementary file 1 — Supplementary material 1. [file 13071_2025_6872_MOESM1_ESM.docx]

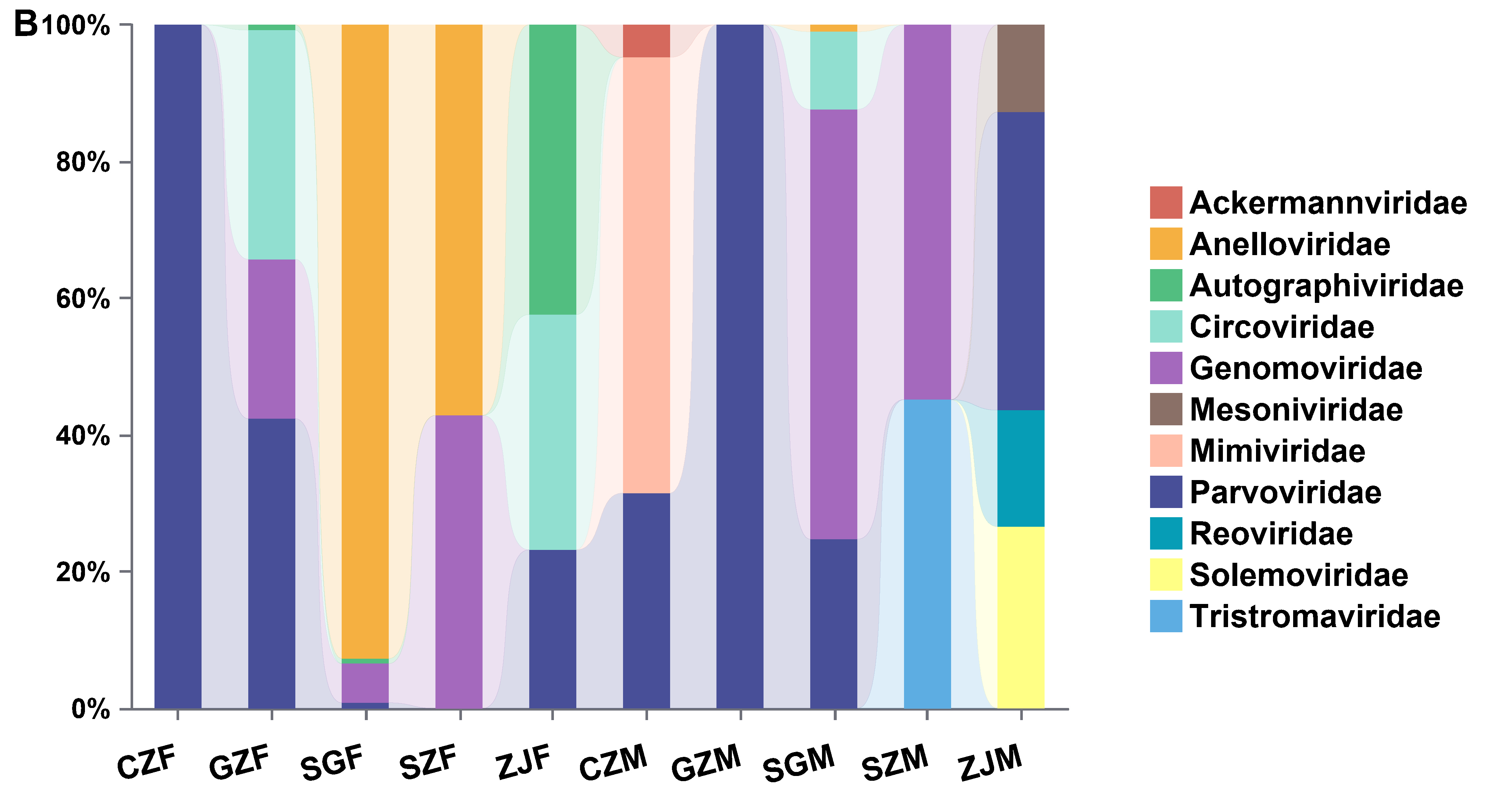

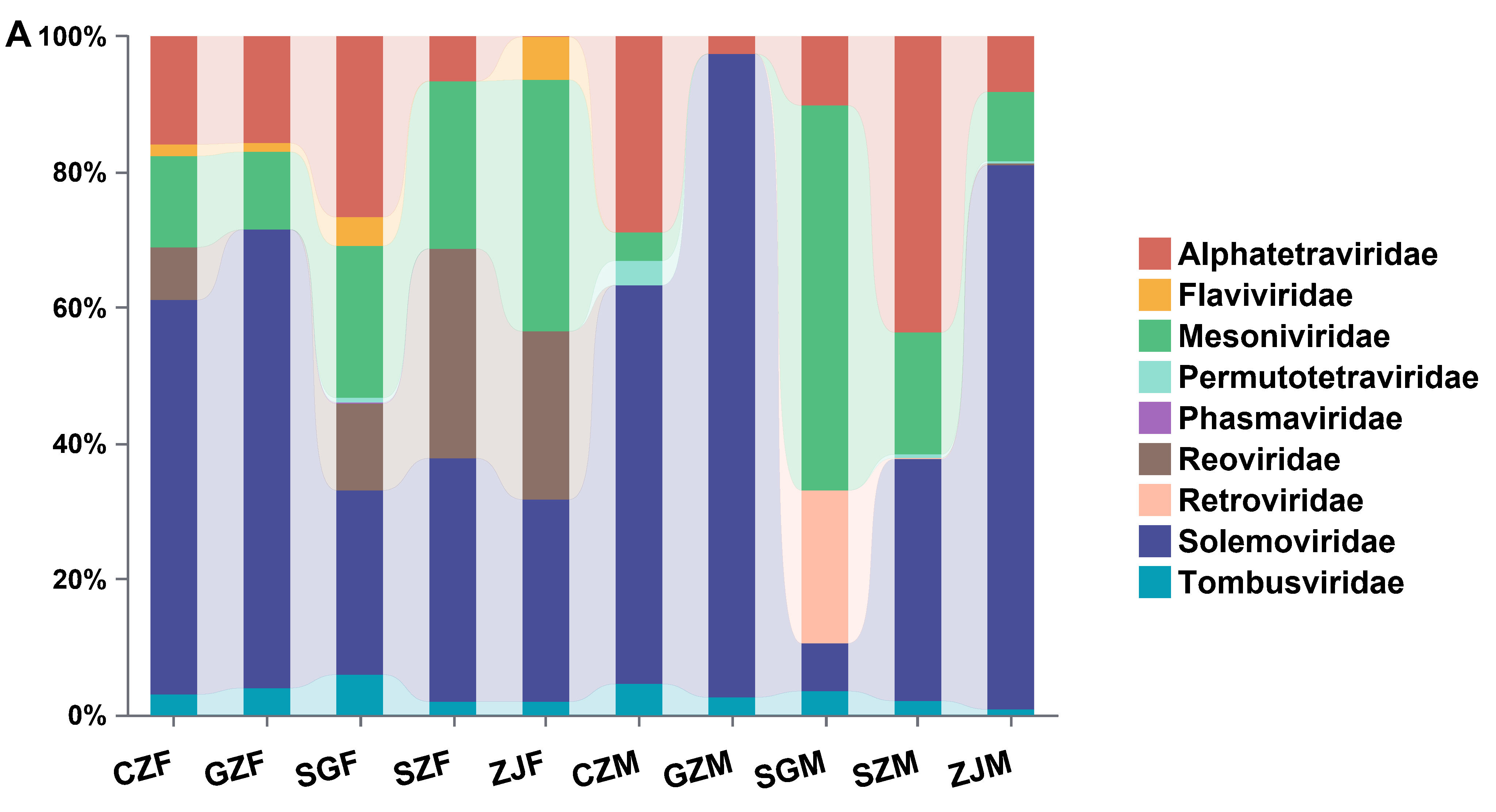
 **Fig.S1** Virome of female *Aedes albopictus* mosquitoes in the five cities of Guangdong Province.

**Fig.S2** Virome of male *Aedes albopictus* mosquitoes in the five cities of Guangdong Province.





**Fig.S3** Geographic-sexual virome clustering in *Aedes Albopictus* DNA-Seq taxonomic profiling.


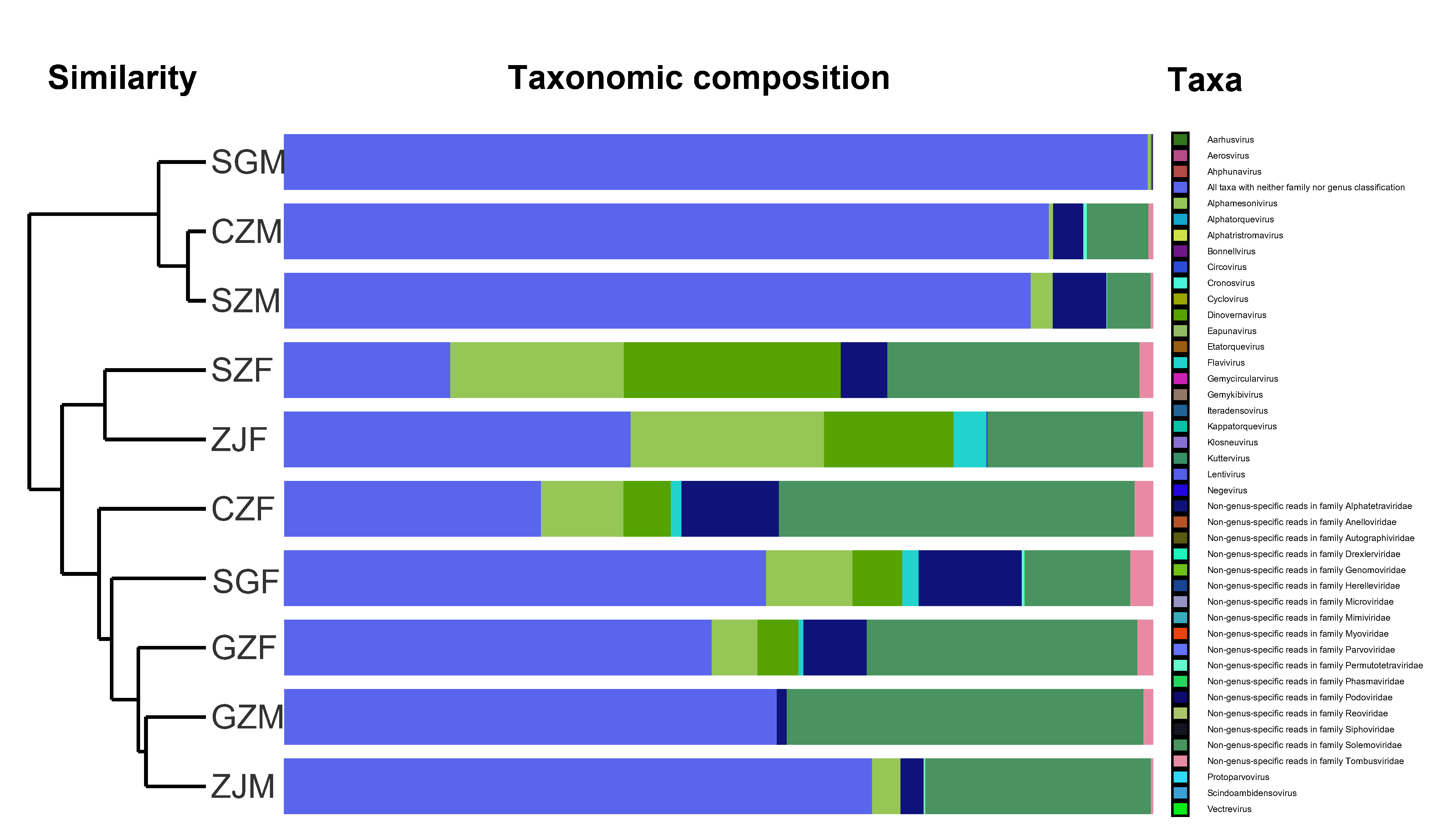
 **Fig.S4** Geographic-sexual virome clustering in *Aedes Albopictus* RNA-Seq taxonomic profiling.
